# Supplementary material for: Trajectories of frailty in aging: Prospective cohort study
Source: PLoS One. 2021 Jul 12;16(7):e0253976. doi: 10.1371/journal.pone.0253976 (PMC8274857; doi:10.1371/journal.pone.0253976)
Supplement: S1 File — (PDF) [file pone.0253976.s001.pdf]

**Supplementary Table 1. Health variables used for construction of cumulative frailty index**

| Sl.no | Variable | Coding |
|---|---|---|
| 1 | Help bathing | Yes = 1, No = 0 |
| 2 | Help dressing | Yes = 1, No = 0 |
| 3 | Help getting in/out of chair | Yes = 1, No = 0 |
| 4 | Help walking around house | Yes = 1, No = 0 |
| 5 | Help eating | Yes = 1, No = 0 |
| 6 | Help grooming | Yes = 1, No = 0 |
| 7 | Help using toilet | Yes = 1, No = 0 |
| 8 | Help up/down stairs | Yes = 1, No = 0 |
| 9 | Help lifting 10 lbs | Yes = 1, No = 0 |
| 10 | Help shopping | Yes = 1, No = 0 |
| 11 | Help with housework | Yes = 1, No = 0 |
| 12 | Help with meal preparations | Yes = 1, No = 0 |
| 13 | Help taking medication | Yes = 1, No = 0 |
| 14 | Help with finances | Yes = 1, No = 0 |
| 15 | Lost more than 10 lbs in last year | Yes = 1, No = 0 |
| 16 | Self rating of health | Poor = 1, Fair = 0.75, Good = 0.5, V. Good = 0.25, Excellent = 0 |
| 17 | How health has changed in last year | Worse = 1, Better/Same = 0 |
| 18 | Hospitalized/ER visits | Yes = 1, No = 0 |
| 19 | Cut down on usual activity (in last month) | Yes = 1, No = 0 |
| 20 | Walk outside | <3 days = 1, ≤ 3 days = 0 |
| 21 | Feel everything is an effort | Most of time = 1, Some time = 0.5, Rarely = 0 |
| 22 | Feel depressed | Most of time = 1, Some time = 0.5, Rarely = 0 |
| 23 | Feel happy | Most of time = 0, Some time = 0.5, Rarely = 1 |
| 24 | Health interfered with social activities | Not at all - Slightly = 0, Moderately - Extremely = 1 |
| 25 | Have trouble getting going | Most of time = 1, Some time = 0.5, Rarely = 0 |
| 26 | Moderate activity affected | Yes = 1, No = 0 |
| 27 | High blood pressure | Yes = 1, No = 0 |
| 28 | Heart attack | Yes = 1, No = 0 |
| 29 | CHF | Yes = 1, No = 0 |
| 30 | Stroke | Yes = 1, No = 0 |
| 31 | Cancer | Yes = 1, No = 0 |
| 32 | Diabetes | Yes = 1, No = 0 |
| 33 | Arthritis | Yes = 1, No = 0 |
| 34 | Chronic lung disease | Yes = 1, No = 0 |
| 35 | Cognitive test: Blessed | <2=0: 2-3=0.25: 4-7=0.50: >7 =1 |
| 36 | Peak flow | Men=<340 liters/min; Women=<310 liters/min |
| 37 | BMI | 1 if BMI <18.5 or >=30 |

| | | |
|---|---|---|
| 38 | Grip strength | Men BMI=<24, GS=<29 : Men BMI 24.1-28, GS=<30:Men BMI>28, GS=<32: Women BMI=<23, GS=<17:Women BMI 23.1-26, GS=<17.3:Women BMI 26.1-29,GS=<18: Women BMI >29, GS=<21 |
| 39 | Falls | Yes = 1, No = 0 |
| 40 | Memory changes | Yes = 1, No = 0 |
| 41 | History of Parkinson's disease | Yes = 1, No = 0 |

**Supplementary table 2. Post hoc analysis of association of frailty trajectories with mortality.** Model adjusted for cohort status and covariates presented below.

| **Variables** | **Hazard ratio (95% confidence intervals)** |
|---|---|
| **Frailty trajectories** | |
| Relatively stable | Reference |
| Mild frail | 2.88 (1.38 - 6.04) |
| Moderate frail | 2.62 (1.08 – 6.36) |
| Severely frail | 5.47 (1.82 – 16.45) |
| Age, years | 1.08 (1.04 – 1.12) |
| Male sex | 2.24 (1.51 – 3.32) |
| Education, years | 0.99 (0.93 – 1.06) |
| Frailty Index score | 1.56 (0.04 – 55.71) |
| Comorbidities score | 1.02 (0.82 – 1.27) |
| Walking speed, cm/s | 0.99 (0.98 – 1.00) |
| Body mass index, $kg/m^2$ | 0.96 (0.92 – 1.01) |
| Free recall score | 1.00 (0.96 – 1.04) |
| DSST score* | 0.81 (0.66 – 1.00) |

***DSST:** Digit symbol substitution test. Scores were standardized as timing of administration varied between LonGenity (90 seconds) and Einstein Aging study (60 seconds).

**Supplementary Table 3. Proteomics and frailty trajectories.** Eleven significant SOMAmer reagent targets associated with frailty trajectories; linear regression comparing the combined frail trajectory groups versus the relatively stable trajectory group as reference.

| SomaId | Target | Target Full Name | UniProt | Estimate | Std Error | p value |
|---|---|---|---|---|---|---|
| SL001774 | H-FABP | Fatty acid-binding protein, heart | P05413 | 0·148 | 0·027 | 6·08E-08 |
| SL008782 | CSPG3 | Neurocan core protein | O14594 | -0·127 | 0·025 | 6·73E-07 |
| SL000498 | Leptin | Leptin | P41159 | 0·267 | 0·054 | 8·62E-07 |
| SL018710 | CA2D3 | Voltage-dependent calcium channel subunit alpha-2/delta-3 | Q8IZS8 | -0·094 | 0·020 | 2·89E-06 |
| SL008968 | DNER | Delta and Notch-like epidermal growth factor-related receptor | Q8NFT8 | -0·094 | 0·020 | 3·39E-06 |
| SL005086 | FABPA | Fatty acid-binding protein, adipocyte | P15090 | 0·126 | 0·027 | 3·85E-06 |
| SL002644 | ERBB1 | Epidermal growth factor receptor | P00533 | -0·053 | 0·011 | 3·9E-06 |
| SL011048 | ANTR2 | Anthrax toxin receptor 2 | P58335 | -0·102 | 0·022 | 6·15E-06 |

| | | | | | | |
|---|---|---|---|---|---|---|
| SL010377 | OMGP | Oligodendrocyte-myelin glycoprotein | P23515 | -0·162 | 0·036 | 7·78E-06 |
| SL004855 | contactin-1 | Contactin-1 | Q12860 | -0·066 | 0·015 | 7·88E-06 |
| SL000070 | Glypican 3 | Glypican-3 | P51654 | -0·101 | 0·023 | 9·08E-06 |

log(SOMAmer) ~ Trajectory Group + Age + Gender + OPEL-OPUS status
